# Heart rate turbulence assessed through ergometry after myocardial infarction: a feasibility study

**DOI:** 10.1590/1516-3180.2021.0884.R1.27012022

**Published:** 2022-08-29

**Authors:** Rafael Alessandro Ferreira Gomes, Dário Celestino Sobral-Filho

**Affiliations:** I MD, MSc, PhD. Attending Physician, Coronary Care Unit, Department of Cardiology, Universidade de Pernambuco (UPE), Recife (PE), Brazil.; II MD, PhD. Associate Professor, Department of Cardiology, Universidade de Pernambuco (UPE), Recife (PE), Brazil.

**Keywords:** Arrhythmias, cardiac, Death, sudden, cardiac, Myocardial infarction, Heart rate turbulence, Cardiac vagal activity, Autonomic dysfunction

## Abstract

**BACKGROUND:**

Coronary artery disease is an important cause of morbidity and mortality. The impact of ventricular arrhythmias with impaired cardiac vagal activity is one of the most recently studied prognostic factors. However, there are no studies evaluating the phenomenon of heart rate turbulence (HRT) during physical exertion.

**OBJECTIVE:**

To study the behavior of HRT during exercise testing, among individuals after myocardial infarction.

**DESIGN AND SETTING:**

Feasibility study conducted in a university hospital among individuals 4-6 weeks after myocardial infarction.

**METHODS:**

All subjects underwent 24-hour Holter monitoring and ergometric stress testing. We considered that abnormal HRT was present if the turbulence onset was ≥ 0% or turbulence slope was ≤ 2.5 mm/relative risk interval.

**RESULTS:**

All 32 subjects were asymptomatic. Their median age was 58 years (interquartile range 12.8) and 70% were male. Abnormal HRT was associated with ventricular dysfunction in this population. We found no differences regarding the behavior of HRT, in relation to age, gender, smoking, systemic arterial hypertension, diabetes mellitus or dyslipidemia. Ergometric stress testing detected premature ventricular beats (PVB) in approximately 44% of the examinations, and these occurred both during the active phase of effort and in the recovery period. The low occurrence of several isolated PVB in beta-blocked subjects made it difficult to perform statistical analysis to correlate HRT between ergometric and Holter testing.

**CONCLUSION:**

The data obtained in this study do not support performing HRT through ergometric stress testing among patients who remain on beta-blockers post-myocardial infarction, for the purpose of assessing cardiac vagal activity.

## INTRODUCTION

Coronary artery disease is a condition characterized by increased atherosclerotic plaque in the epicardial arteries and is associated with high morbidity and mortality. Coronary artery disease accounts for nearly 360,000 events per year^1^ in the United States, among which most occur in the hospital setting, and many events evolve to death before the patients are transported to the emergency room.^1^ In the first six months of 2019, 137,713 hospitalizations due to coronary artery disease were recorded in Brazil, and 5.8% culminated in in-hospital death.^2^

Some clinical factors and complementary test markers help in the prognostic evaluation of coronary artery disease. Among these, the following can be highlighted: advanced age, male gender, systemic arterial hypertension, diabetes mellitus, dyslipidemia, smoking and myocardial dysfunction.^3^ ^,^ ^4^ However, the impairment of cardiac vagal activity over the first year after a diagnosis of myocardial infarction has been made is also a good indicator for identifying the development of heart disease and sudden death over the short and medium term.^5^ ^,^ ^6^ ^,^ ^7^

Analysis on the behavior of heart rate turbulence (HRT), obtained through 24-hour Holter monitoring, is one of the easiest and most efficient means for assessing cardiac autonomic dysfunction.^8^ Sade et al.^8^ found that HRT was similar to the ejection fraction in an assessment of the prognosis of 128 individuals post-infarction. On the other hand, the Innovative Stratification of Arrythmic Risk - Heart Rate Turbulence (ISAR-HRT)^9^ study showed that altered HRT parameters increased the risk of death almost sixfold, in a prospective analysis on 1,500 survivors of myocardial infarction analyzed over a 22-month period. This risk exceeded the risks attributed to severe ventricular dysfunction, diabetes mellitus and age over 65 years.

The low clinical use of HRT as a risk predictor, which was first put forward by Schmidt et al.,^5^ may be related to the low sensitivity that it has been perceived to have in some studies.^8^ It may also be because 24-hour Holter monitoring is not routinely indicated after myocardial infarction, considering that HRT is determined and analyzed in Holter monitoring.^10^

Ergometric stress testing assists in risk assessments on coronary events through analysis on clinical, electrocardiographic and hemodynamic parameters. Modulation of autonomous tonus takes place during physical exertion, which gives rise to increased sympathetic activity during the active phase of effort, while cardiac vagal activity increases in the recovery period.^11^ Thus, vagal activity may increase during physical exercise. Hence, ergometric stress testing is already incorporated in regular monitoring for patients with coronary artery disease.

## OBJECTIVE

The aim of our study was to evaluate the behavior of post-myocardial infarction HRT during ergometric stress testing.

## METHODS

This was an observational, prospective study using primary data to estimate changes in HRT during ergometric stress testing among individuals who had recently had a myocardial infarction episode. The study was conducted in the “Professor Luiz Tavares” Cardiological Emergency Service (Pronto Socorro Cardiológico Universitário de Pernambuco Professor Luiz Tavares), which is affiliated with the University of Pernambuco, between 2018 and 2019. All the patients met the criteria for myocardial infarction, in accordance with the fourth universal definition of myocardial infarction.^12^ We excluded individuals with a history of previous events relating to coronary disease, those who could not undergo the ergometric stress testing (due to orthopedic/neurological problems, balance deficits or peripheral vascular alterations) and those who presented factors that precluded the possibility of HRT (atrial fibrillation, cardiac pacemaker and artifacts in the examination recordings).

All of the individuals included in this study had become asymptomatic by the time that they reached four to eight weeks after the ischemic event and they were continuing to use of beta-blockers regularly. All of them underwent 24-hour Holter monitoring (CardioLight 3-channel recorder; Cardios, São Paulo, Brazil) and ergometric stress testing using the Naughton protocol (KT 10200 AT multi-programmable treadmill; Inbramed, Porto Alegre, Brazil). Turbulence onset (TO) ≥ 0% or turbulence slope (TS) ≤ 2.5 mm/relative risk interval in 24-hour Holter monitoring was considered to be the gold standard for abnormal HRT.

The analysis on the HRT parameters was standardized in accordance with the study by Bauer et al.^11^ In order to eliminate errors in the analysis, we excluded the following: interpolated premature ventricular beats (PVBs); PVB with prematurity less than 20%; PVB with compensation pause below 120% of the average of the last five previous relative risk (RR) or PVB values; and very short (< 300 ms) or very long (> 2000 ms) PVB tachograms.

TO was calculated based on the last two sinus RR intervals immediately before the PVB-coupling interval, and the two sinus RR intervals immediately after the compensatory pause. TO (as a percentage) has negative values for patients with low cardiovascular risk since there is an immediate heart rate acceleration after PVB.

$$Turbulence\;onset=\frac{\left({RR}_{1}+{RR}_{2}\right)-\left({RR}_{-1}+{RR}_{-2}\right)}{\left({RR}_{2}+{RR}_{1}\right)}\times 100(\%)$$

Where RR = R-to-R wave interval in electrocardiogram

On the other hand, TS was calculated using the slope of the line formed by five RR intervals after the PVB, which were obtained from among the first 15 sinus RR intervals that followed the PVB. TS is expressed in milliseconds per RR interval, and the heart rate of patients with a low cardiovascular risk is decreased by up to 8 beats/min following the initial acceleration caused by the PVB. Thus, the reference value is > 2.5 ms/RR, which translates as the maximum variation in sinus RR intervals (ms) among the five sinus RR intervals to be analyzed.

$$Turbulence\;slope=\frac{Y5-Y1}{X5-X1}$$

Where Y = maximum positive regression slope assessed after the PVB; and X = five consecutive sinus rhythm R-R intervals after PVB.

The choice between parametric and nonparametric tests was made according to the presence or absence of normal distribution of the data, as shown in the Kolmogorov-Smirnov test. Parametric continuous variables were expressed as the mean ± standard deviation, and nonparametric variables as the median and interquartile range. The Mann-Whitney or Student t test was used, as indicated. Strategic variables were calculated as relative and absolute frequencies. A significance level of P < 0.05 and a statistical power of 80% were adopted for all tests. The IBM SPSS version 21.0 software (IBM, Chicago, Illinois, United States) was used for the statistical analysis.

This study was approved by the Research Ethics Committee of the Hospital Universitário Oswaldo Cruz *-* Pronto-Socorro Cardiológico Universitário de Pernambuco (HUOC-PROCAPE), under registration number 2.681.495 of May 29, 2018.

## RESULTS

The population had a median age of 58 years (interquartile range, IQR 12.8) and about two-thirds were male. Among the 32 individuals evaluated for the presence of HRT, 24 presented normal TO and TS parameters and eight individuals presented abnormal TO and/or TS. We did not find any differences between the normal and abnormal HRT groups regarding cardiovascular risk factors (presence of hypertension, diabetes mellitus, dyslipidemia or smoking), ischemic presentation (ST-segment-elevation or non-ST-segment-elevation infarction), infarct wall affected (anterior or inferior) or ventricular depolarization (QRS) complex duration. The parameters of heart rate variability were similar in the groups that presented normal HRT and abnormal HRT.

The individuals with abnormal HRT showed an association with lower left ventricular ejection fraction, compared with those with normal HRT (46.6% versus 58.6%; P = 0.004). The demographic and baseline characteristics of the patients are summarized in Table 1.

**Table 1.** Demographic and cardiovascular profile of the study population

Variable	Total (n = 32)	Normal HRT (n = 24)	Abnormal HRT (n = 8)	P
Age, median (IQR), years	58.0 (12.8)	58.0 (11.5)	59.0 (20.5)	0.535
Gender				
Male, n (%)	22 (68.8%)	15 (68.2%)	7 (31.8%)	0.186
Female, n (%)	10 (31.2%)	9 (90%)	1 (10%)	
Smoking				
Yes, n (%)	3 (9.4%)	2 (66.7%)	1 (33.3%)	0.726
No, n (%)	29 (90.6%)	22 (75.9%)	7 (24.1%)	
Systemic arterial hypertension				
Yes, n (%)	22 (68.8%)	18 (81.8%)	4 (18.2%)	0.186
No, n (%)	10 (31.2%)	6 (60%)	4 (40%)	
Diabetes mellitus				
Yes, n (%)	9 (28.1%)	8 (88.9%)	1 (11.1%)	0.256
No, n (%)	23 (71.9%)	16 (69.6%)	7 (30.4%)	
Dyslipidemia				
Yes, n (%)	15 (46.9%)	11 (73.3%)	4 (26.7%)	0.838
No, n (%)	17 (53.1%)	13 (76.5%)	4 (23.5%)	
LVEF	55.7 ± 11.1	58.6 ± 9.03	46.6 ± 10.6	0.004
QRS complex length (ms)	89.5 ± 16.8	89.0 ± 13.6	96.0 ± 31.7	0.448
Heart rate variability				
SDNN (ms), mean ± SD%	119.7 ± 40.7	118.5 ± 39.1	107.3 ± 39.2	0.491
RMSSD (ms), mean ± SD	39.3 ± 35.1	41.4 ± 36.4	25.7 ± 12.9	0.247
pNN50 (%), mean ± SD	7.8 ± 7.0	7.6 ± 7.2	5.0 ± 5.2	0.363
Myocardial infarction type				
STEMI (%), mean ± SD	26 (81.3%)	20 (76.9%)	6 (23.1%)	0.601
NSTEMI (%), mean ± SD	6 (18.8%)	4 (66.7%)	2 (33.3%)	
Damaged myocardial wall				
Previous (%), mean ± SD	14 (53.8%)	9 (64.3%)	5 (35.7%)	0.240
Inferior (%), mean ± SD	12 (46.2%)	11 (91.7%)	1 (8.3%)	

IQR = interquartile range; LVEF = left ventricle ejection fraction; QRS = ventricular depolarization; SDNN = mean of the standard deviations of all normal sinus RR intervals for all 5-min segments; RMSSD = root mean square of successive differences between normal heartbeats; pNN50 = proportion of NN50 divided by the total number of NN (R-R) intervals.; STEMI = ST-segment-elevation myocardial infarction; NSTEMI = non-ST-segment-elevation myocardial infarction; SD = standard deviation; HRT = heart rate turbulence.

All ergometric stress testing was stopped when fatigue was reached or upon reaching the submaximal heart rate. The mean baseline heart rate was 66.5 ± 13.2 beats per minute (bpm) and the mean peak heart rate was 123.2 ± 21.2 bpm. The patients achieved an estimated metabolic equivalent performance of 5.94 ± 2.27 metabolic equivalents (MET). The hemodynamic changes in pressure levels were compatible with the degree of exertion performed. Ventricular extrasystoles were detected in approximately 44% of the examinations and occurred both during the active phase of effort and in the recovery period (Table 2).

**Table 2.** Ergometric parameters of the study population

Variable	Total (n = 32)	Normal HRT (n =24)	Abnormal HRT (n = 8)	P
Baseline HR (bpm), mean ± SD, %	66.51 ± 13.2	65.47 ± 14.4	77.0 ± 13.2	0.064
Peak HR (bpm), mean ± SD, %	123.2 ± 21.2	120.3 ± 22.3	129.7 ± 20.7	0.319
HR recovery after 1 min (bpm), mean ± SD, %	16.7 ± 9.8	15.8 ± 9.9	16.6 ± 11.7	0.870
MET, mean ± SD	5.94 ± 2.27	6.01 ± 2.51	5.21 ± 1.91	0.427
PVB, n (%)	14 (43.8%)	11 (78.6%)	3 (21.4%)	0.681

HR = heart rate; SD = standard deviation; min = minute; MET = metabolic equivalent; PVB = premature ventricular beat; bpm = beats per minute; HRT = heart rate turbulence.

Only three individuals had more than five isolated PVBs during the ergometric stress testing (minimum number required to perform the HRT parameter calculations, according to Bauer et al.).^11^ This extremely low number did not allow us to undertake any statistical treatment of associations of HRT parameters between the ergometric test and 24-hour Holter test.

## DISCUSSION

In our study, it was not possible to adequately perform analysis on HRT using ergometric stress testing due to the low density of ventricular arrhythmia. All the individuals analyzed were making regular use of beta-blockers, and this medication is known to significantly decrease the occurrence of PVBs.

The physiological mechanism of HRT involves patency of baroreceptors and the autonomic nervous system. Thus, a PVB is expected to promote a transient hemodynamic change, initially manifested as a decrease in the systolic volume perceived in the baroreceptors. Since blood pressure is the product of ejected systolic volume, heart rate and peripheral vascular resistance, the immediate response is inhibition of vagal stimuli with a consequent increase in blood pressure levels. Therefore, there should be an accelerated heart rate and increased peripheral vascular resistance after a PVB^10^ in individuals without cardiac dysautonomia.

This first response is usually ephemeral, since baroreceptors will also detect an elevation in blood pressure and consequently recruit the vagal stimulus that is responsible for heart rate deceleration.^10^ Later on, they will return to the hemodynamic situation prior to PVBs.

Several studies have demonstrated the prognostic importance of HRT, post-myocardial infarction. Hoshida et al. analyzed 313 patients after myocardial infarction, over a mean follow-up of three years, and showed that HRT parameters were strong predictors of cardiac mortality (heart rate, HR 5.7; 95% confidence interval, CI 2.1-15.9; P = 0.0008).^13^ Huikuri et al. followed up 310 patients who had suffered myocardial infarction two years earlier and found that TS was associated with ventricular arrhythmia (HR 2.8; 95% CI 1.1-7.2; P = 0.038).^14^

In the ISAR-HRT study, 1,500 survivors of acute myocardial infarction were assessed. It was found that combination of abnormal TO and TS was the strongest predictor of mortality (odds ratio 5.9; 95% CI 2.9-12.2). Another important finding of that study was that HRT was able to provide information on mortality risk in a more relevant way than that obtained using the left ventricle ejection fraction (LVEF).^9^

In the REFINE study, on 320 patients with acute myocardial infarction and LVEF of < 40%, HRT proved to be a good predictor of cardiac death or resuscitated cardiac arrest (HR 2.91; 95% CI 1.13-7.48; P = 0.026) when at least one of the HRT parameters was altered.^15^

The few studies^16^ ^,^ ^17^ that have attempted to analyze the behavior of HRT over short periods failed to show good accuracy because they were conducted at rest and expected the individuals to spontaneously present an isolated PVB. For example, the Multicenter Automatic Defibrillator Implantation Trial II (MADIT II) study evaluated the behavior of HRT among approximately 900 patients after recent myocardial infarction and found that there was no statistically significant association between HRT parameters and mortality, from at-rest records over 10 minutes of recording.

Some efforts have been made to spread the concept of the prognostic value of HRT through faster recordings. Thus, physical exertion could be used as an alternative for obtaining this parameter by inducing ventricular arrhythmia in the active phase and increasing cardiac vagal activity in recovery. Ergometric stress testing is already envisaged in the follow-up of coronary artery disease patients and, thus, if it is possible to analyze the parameters of HRT during ergometric stress testing, this might form a valuable piece of information for these individuals.

In our study, heart rate variability parameters were unchanged in both the normal and the abnormal HRT groups. Suspension of the patients’ use of beta-blockers could have sensitized the examination, thereby providing an increase in the occurrence of ventricular arrhythmia and enabling detection of changes in cardiac vagal tone. However, for ethical reasons, suspension of this medication was not authorized, even if it would have only been temporary.

The major limitation of our study was the fact that the small sample size precluded observation of any statistical difference regarding the behavior of HRT involving demographic data and cardiovascular risk factors. Likewise, this did not enable assessment of the possible association of the HRT parameters obtained through ergometry with the gold standard obtained through the 24-hour Holter monitoring.

## CONCLUSION

The data obtained in this study do not support assessment of HRT through ergometric stress testing in patients who remain on beta-blockers post-myocardial infarction, for the purpose of assessing cardiac vagal activity, as a replacement for 24-hour Holter monitoring.

## References

[1] Benjamin EJ, Muntner P, Alonso A (2019). Heart Disease and Stroke Statistics-2019 Update: A Report From the American Heart Association. Circulation.

[2] Ministério da Saúde. DATASUS Transformação Digital para o SUS.

[3] Johansson S, Rosengren A, Young K, Jennings E (2017). Mortality and morbidity trends after the first year in survivors of acute myocardial infarction: a systematic review. BMC Cardiovasc Disord.

[4] Asaria P, Elliott P, Douglass M (2017). Acute myocardial infarction hospital admissions and deaths in England: a national follow-back and follow-forward record-linkage study. Lancet Public Health.

[5] Schmidt G, Malik M, Barthel P (1999). Heart-rate turbulence after ventricular premature beats as a predictor of mortality after acute myocardial infarction. Lancet.

[6] Disertori M, Masè M, Rigoni M, Nollo G, Ravelli F (2016). Heart Rate Turbulence is a Powerful Predictor of Cardiac Death and Ventricular Arrhythmias in Postmyocardial Infarction and Heart Failure Patients: A Systematic Review and Meta-Analysis. Circ Arrhythm Electrophysiol.

[7] Carney RM, Howells WB, Blumenthal JA (2007). Heart rate turbulence, depression, and survival after acute myocardial infarction. Psychosom Med.

[8] Sade E, Autemir K, Oto A (2003). Assessment of heart rate turbulence in the acute phase of myocardial infarction for long-term prognosis. Pacing Clin Electrophysiol.

[9] Barthel P, Schneider R, Bauer A (2003). Risk stratification after acute myocardial infarction by heart rate turbulence. Circulation.

[10] Bauer A, Malik M, Schmidt G (2008). Heart rate turbulence: standards of measurement, physiological interpretation, and clinical use: International Society for Holter and Noninvasive Electrophysiology Consensus. J Am Coll Cardiol.

[11] Bauer A, Malik M, Barthel P (2006). Turbulence dynamics: an independent predictor of late mortality after acute myocardial infarction. Int J Cardiol.

[12] Thygesen K, Alpert JS, Jaffe AS (2019). Fourth universal definition of myocardial infarction (2018). Eur Heart J.

[13] Hoshida K, Miwa Y, Miyakoshi M (2013). Simultaneous assessment of T-wave alternans and heart rate turbulence on holter electrocardiograms as predictors for serious cardiac events in patients after myocardial infarction. Circ J.

[14] Huikuri HV, Raatikainen MJ, Moerch-Joergensen R (2009). Prediction of fatal or near-fatal cardiac arrhythmia events in patients with depressed left ventricular function after an acute myocardial infarction. Eur Heart J.

[15] Exner DV, Kavanagh KM, Slawnych MP (2007). Noninvasive risk assessment early after a myocardial infarction the REFINE Study. J Am Coll Cardiol.

[16] Berkowitsch A, Zareba W, Neumann T (2004). Risk stratification using heart rate turbulence and ventricular arrhythmia in MADIT II: usefulness and limitations of a 10-minute holter recording. Ann Noninvasive Electrocardiol.

[17] Steger A, Müller A, Barthel P (2019). Polyscore of Non-invasive Cardiac Risk Factors. Front Physiol.

